# Ecological and Human Health Risk Assessment of Metals in Peruvian Avocados Using a Probabilistic Approach

**DOI:** 10.3390/foods15010082

**Published:** 2025-12-26

**Authors:** Myryam Yoplac-Navarro, Dorila E. Grandez-Yoplac, Pablo Rituay, Jonathan Alberto Campos Trigoso, Ligia García, Erick Arellanos, Jorge Enrique Ortiz-Porras, Grobert A. Guadalupe

**Affiliations:** 1 Facultad de Ingeniería Industrial, Universidad Nacional Mayor de San Marcos, Lima 15081, Peru; myryam.yoplac@unmsm.edu.pe (M.Y.-N.); jortizpo@unmsm.edu.pe (J.E.O.-P.); 2 Grupo de Investigación en Seguridad Alimentaria (GISA), Instituto de Investigación, Innovación y Desarrollo para el Sector Agrario y Agroindustrial (IIDAA), Universidad Nacional Toribio Rodríguez de Mendoza de Amazonas, Calle Higos Urco N° 342-350-356—Calle Universitaria N° 304, Chachapoyas 01001, Peru; dorila.grandez@untrm.edu.pe; 3 Centro de Investigación Economía Circular y Prospectiva de Agronegocios, Instituto de Investigación en Negocios Agropecuarios, Facultad de Ingeniería Zootecnista, Biotecnología, Agronegocios y Ciencia de Datos, Universidad Nacional Toribio Rodríguez de Mendoza de Amazonas, Chachapoyas, Chachapoyas 01001, Peru; pablo.rituay@untrm.edu.pe; 4 Escuela de Posgrado, Programa Doctoral en Ciencias para el Desarrollo Sustentable, Facultad de Ingeniería Zootecnista, Biotecnología, Agronegocios y Ciencia de Datos, Universidad Nacional Toribio Rodríguez de Mendoza de Amazonas, Chachapoyas 01001, Peru; 5 Facultad de Ingeniería Zootecnista, Biotecnología, Agronegocios y Ciencia de Datos (FIZBAC), Universidad Nacional Toribio Rodríguez de Mendoza de Amazonas, Chachapoyas 01001, Peru; ligia.garcia@untrm.edu.pe; 6 Grupo de Investigación en Valoración Económica de la Biodiversidad (VEB), Universidad Nacional Toribio Rodríguez de Mendoza de Amazonas, Calle Higos Urco N° 342-350-356—Calle Universitaria N° 304, Chachapoyas 01001, Peru; erick.arellanos@untrm.edu.pe; 7 Instituto Universitario de Ingeniería de Alimentos Food-UPV, Universitat Politècnica de València, Camino de Vera s/n, 46022 Valencia, Spain

**Keywords:** food safety, value chain, risk assessment, health, chemical hazard, agribusiness

## Abstract

This study evaluated the ecological and health risks associated with metals in Peruvian avocado cultivation from a One Health perspective. Between January and September 2025, a total of 190 soil and fruit samples were collected from major producing regions (Amazonas, Áncash, Ayacucho, Cusco, Huancavelica, Ica, La Libertad, and Lima) to quantify arsenic (As), cadmium (Cd), chromium (Cr), mercury (Hg), nickel (Ni), and lead (Pb) using microwave plasma atomic emission spectrometry (MP-AES). Results showed regional variability in soil metal concentrations, with higher As (76.17 ± 17.35 mg/kg), Cd (0.55 ± 1.04 mg/kg), and Pb (25.35 ± 6.02 mg/kg). Cr concentrations in avocados were below the detection limit (<0.003 mg/kg), while As (<0.003–0.192 mg/kg), Cd (<0.005–0.130 mg/kg), Hg (<0.005–0.428 mg/kg), Ni (<0.005–0.172 mg/kg), and Pb (<0.005–0.396 mg/kg) exhibited broader concentration ranges. Bioaccumulation (BAF) values < 1 confirmed low translocation. The geo-accumulation index (Igeo) and ecological risk (ER) indicated uncontaminated or moderately contaminated soils with low ecological risk. In terms of health risk, the hazard quotient (HQ) and hazard index (HI) were <1, representing a low level of concern for non-genotoxic effects. The cancer risk (CR) values for both metals ranged from 10^−8^ to 10^−5^, indicating a non-significant carcinogenic risk for Pb (<10^−6^) and an acceptable risk for Cd (10^−4^).

## 1. Introduction

The avocado (*Persea americana*) is a major crop in the global tropical fruit market. Its production is projected to reach 12 million tons by 2030, over three times the volume reported in 2010, driven by rising international demand and strong export prices [1]. From a nutritional perspective, avocados stand out for their high nutrient density, particularly in their content of monounsaturated fatty acids, such as oleic acid, as well as dietary fiber, vitamins E, C, and K, and essential minerals [2,3]. It also contains bioactive compounds, such as carotenoids, tocopherols, and phenolics, which contribute to its notable antioxidant capacity [4]. Regular avocado consumption has been associated with significant improvements in serum lipid profiles, including increases in High-Density Lipoprotein (HDL) cholesterol and reductions in Low-Density Lipoprotein (LDL) cholesterol and triglycerides. These metabolic effects are associated with a lower incidence of cardiovascular diseases and improved body weight regulation [5,6]. Consequently, avocado is recognized as a strategic food for the prevention of cardiometabolic disorders and the promotion of a healthy diet [7,8].

Peru ranked as the world’s second-largest exporter of fresh avocado in 2024 and the third most important agro-export product, with exports reaching USD 1.247 billion and 570,407 tons, according to data from the National Superintendency of Customs and Tax Administration (SUNAT). In Peru, avocado production encompasses a diverse range of cultivated varieties, including Criollo, Dedo, Ettinger, Fuerte, Hall, Hass, Naval, Queen, Villa Campa, and Zutano. La Libertad is the main producing area, followed by Ica and Lima [9]. Sustaining this agro-export leadership requires strict compliance with food safety, traceability, and environmental sustainability standards. However, the accumulation of metals in soils used for avocado cultivation poses a significant challenge. In 2025, the European Union’s Rapid Alert System for Food and Feed (RASFF) reported nine alerts for Peruvian avocados that exceeded the maximum cadmium (Cd) limit of 0.05 mg/kg, resulting in the withdrawal of several batches in European countries. Similar notifications have been reported in other exporting nations, such as Colombia and the Dominican Republic, due to comparable cadmium exceedances in their avocado exports [10].

Peru is one of the leading mining countries in Latin America, with a strong presence in the production of gold, lead, and tin, and ranking second in silver, zinc, and copper output [11]. Several studies have shown that the country’s mining tailings contain high concentrations of metals, posing potential risks to soil and water resources [12,13]. In agriculture, it has been documented that certain fertilizer and pesticide formulations contain metals such as arsenic, mercury, copper, lead, chromium, zinc, aluminum, lithium, boron, barium, and titanium [14,15,16,17,18,19,20].

Heavy metals, due to their toxicity, persistence, and bioaccumulation potential, pose environmental and health threats even at low concentrations [21,22,23]. Although their natural presence in soil is limited, anthropogenic activities such as agriculture, industry, and mining increase their levels, deteriorating the chemical, physical, and biological properties of soils [24,25,26]. This increase facilitates their absorption by roots and translocation within plants, affecting essential physiological processes and reducing agricultural productivity [27,28,29,30,31]. Plant roots are the first tissues to absorb available metals from the soil, which are subsequently translocated to the stem, leaves, fruits, and seeds [32,33,34,35]. The accumulation of these metals in the edible parts of crops introduces potential health risks, as elements such as arsenic (As), cadmium (Cd), chromium (Cr), mercury (Hg), nickel (Ni), and lead (Pb) exhibit genotoxic, carcinogenic, and non-genotoxic effects on vital systems [36,37,38,39,40]. Under the One Health approach, which recognizes the interdependence of human, animal, and environmental health, global coordinated action is promoted through the Joint Plan of Action 2022–2026, led by international organizations [41,42].

Despite progress in the environmental monitoring of potentially toxic elements and their ecological and health effects, significant gaps remain regarding their actual transfer to fruits and the variability among producing regions, particularly in the case of Peruvian avocados. While previous studies in the country have addressed soil and water contamination associated with mining activities [43,44,45] and their impacts on crops, such as coffee [11], there is a lack of information on the bioaccumulation of metals in avocados and on probabilistic risk assessments related to their consumption.

In this context, the present study addresses a critical knowledge gap through an integrated approach grounded in the One Health paradigm, aiming to evaluate the ecological and health risks associated with the presence of As, Cd, Cr, Hg, Ni, and Pb in Peruvian avocado production systems, considering regional variability from a probabilistic perspective. To this end, metal concentrations were determined in soil and fruit samples from the regions of Amazonas, Áncash, Ayacucho, Cusco, Huancavelica, Ica, La Libertad, and Lima. Evaluation indicators, including bioaccumulation factors, the geo-accumulation index, individual and potential ecological risk, as well as the hazard index and carcinogenic risk to humans, were applied.

## 2. Materials and Methods

### 2.1. Sample Collection and Pre-Treatment

**Figure 1:**
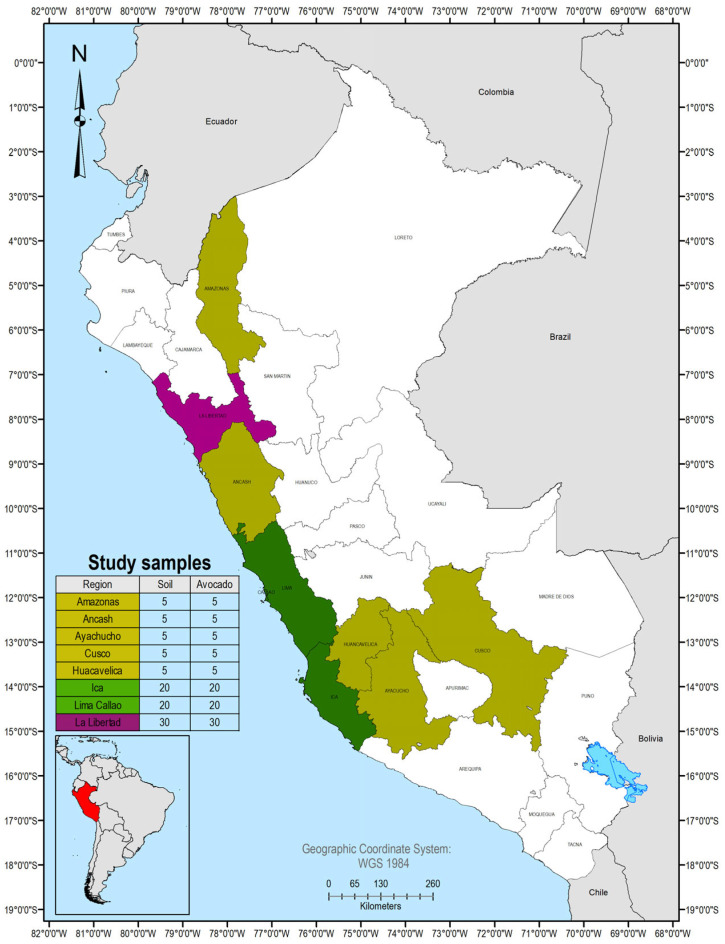
Map of Peru showing the sampling sites.

Between January and September 2025, in Peru, a total of 190 samples were collected from the following regions: Amazonas (10), Áncash (10), Ayacucho (10), Cusco (10), Huancavelica (10), Ica (40), La Libertad (60), and Lima (40). In each region, both soil and avocado samples were obtained (Figure 1).

Soil samples were randomly collected from the top layer (3–20 cm) using a stainless-steel spoon. Each composite sample consisted of three thoroughly mixed subsamples. Soil samples were dried at 60 °C, ground, and thoroughly mixed before sieving through a 0.15 mm nylon mesh. The peel of each avocado fruit was removed, and the pulp was individually cut into thin slices (approximately 2–3 mm thick) using a stainless-steel knife. Avocado samples were dried at 70 °C for 24 h (Model SN260, Memmert GmbH + Co. KG, Schwabach, Germany), ground in a laboratory mill (Model MP-100, Trittón Perú, Lima, Peru), and homogenized in a metal-free mortar (Model EIS-7115, Eisco Scientific, Mumbai, India). All samples were collected in polypropylene bags, were transported at 4 °C, and stored at −20 °C until subsequent laboratory analysis.

The samples were subjected to acid digestion. A 0.5 g sieved sample was placed in digestion vessels under a fume hood. A total of 5 mL of HNO_3_ was added to each sample and they were heated in a HotBlock system at 95 ± 5 °C for 15 min. After cooling, an additional 2.5 mL of concentrated HNO_3_ was added, and heating was continued for 30 min, repeating the procedure until the brown fumes were eliminated. Digestion was maintained for a maximum of 2 h or until the volume was reduced to approximately 5 mL, avoiding complete evaporation. Subsequently, 1 mL of distilled water and 1.5 mL of H_2_O_2_ were added, allowing the mixture to react for 5–10 min before reheating. When necessary, aliquots of 0.5 mL of H_2_O_2_ (up to a total of 5 mL) were added until the sample’s color stabilized. If intense effervescence occurred, the temperature was reduced to control the reaction. Finally, 5 mL of concentrated HCl was added, and the mixture was heated again to 95 °C for 15 min. After cooling, the digestate was transferred to a 250 mL volumetric flask, made up with distilled water, and filtered through Whatman No. 44 paper (Whatman™, Cytiva, Little Chalfont, UK). The solutions obtained were ready for quantification.

### 2.2. Chemical Analysis

The metals As, Cd, Cr, Hg, Ni, and Pb were analyzed at the Soil and Water Research Laboratory (LABISAG) of the National University Toribio Rodríguez de Mendoza of Amazonas, Peru, which is accredited by the National Institute for Quality of Peru (INACAL) under ISO/IEC 17025:2017. Metal concentrations were measured using microwave plasma atomic emission spectroscopy (MP-AES) with an inductively coupled plasma (ICP) method, employing an Agilent 4100 MP-AES spectrometer (Agilent Technologies, Santa Clara, CA, USA) equipped with a standard torch, an Inert One Neb nebulizer, and a double-pass glass cyclonic spray chamber from Agilent Technologies. The determination method is described in Guadalupe et al. [23].

The limits of quantification (LOQ) and detection (LOD) were established based on the minimum detectable amount of each analyte, defined by a signal-to-noise ratio of 3:1 for LOD and 10:1 for LOQ [46]. These limits are expressed in terms of concentration (mg/kg). The following wavelengths were selected for the quantification of As, Cd, Cr, Hg, Ni, and Pb: 228.802 nm for As, 405.781 nm for Cd, 193.695 nm for Cr, 425.433 nm for Hg, 214.160 nm for Ni, and 253.652 nm for Pb. Instrument calibration was performed using standard solutions of As, Cd, Cr, Hg, Ni, and Pb at variable concentrations, all prepared from a 1000 mg/kg stock standard solution. Analytical quality for detection and quantification was verified using blank and duplicate samples. Parameter validation was performed with ten replicates, as reported in Table S1.

### 2.3. Data Analysis

The ability of plants to absorb and accumulate metals from the soil was determined using the Bioaccumulation Factor (BAF). The level of soil contamination was estimated through the Geo-accumulation Index (Igeo). Likewise, the ecological risk in the environment was assessed using the Ecological Risk Index (ER) and the Potential Ecological Risk Index (RI). These indicators were applied to the analyzed soils [47,48,49,50,51,52,53], and the corresponding equations are presented in Table 1.

The health risk assessment was conducted using a probabilistic approach with the @Risk software, version 8 (Palisade, Newfield, NY, USA). The Hazard Quotient (HQ) was calculated to estimate the likelihood that a contaminant may cause non-genotoxic effects on human health, while the Hazard Index (HI) corresponded to the sum of all HQ values obtained [60,63,64]. Regarding genotoxic effects, the Cancer Risk (CR) was estimated to quantify the associated hazard [65,66,67].

To incorporate the variability and uncertainty inherent in the input data, the concentrations of heavy metals detected in soil and avocado samples from each region were fitted to specific probability density functions. The results for each parameter were obtained by applying a Monte Carlo simulation combined with Latin Hypercube sampling, using ten runs of 100,000 iterations. This procedure generated a probability density function for each analyzed indicator. The obtained concentrations of As, Cd, Cr, Hg, Ni, and Pb in soil and avocado samples are presented in Section 3.1.

## 3. Results and Discussion

### 3.1. Concentration of Metals

Table 2 presents the concentrations of As, Cd, Cr, Hg, Ni, and Pb, expressed in mg/kg, determined in both agricultural soils and avocado fruits from eight regions of Peru: Amazonas, Áncash, Ayacucho, Cusco, Huancavelica, Ica, La Libertad, and Lima.

#### 3.1.1. Concentration of Metals in Soil

The results revealed regional heterogeneity in metal concentrations, with predominantly moderate and locally elevated levels depending on the specific metal. As concentrations ranged from 28.04 to 99.01 mg/kg. The highest concentrations were observed in Lima (76.17 ± 17.35 mg/kg) and Ayacucho (58.98 ± 7.62 mg/kg), while the lowest values were found in Huancavelica (34.01 ± 6.50 mg/kg) and Amazonas (42.41 ± 8.21 mg/kg). The highest As levels recorded in Áncash (52.47 mg/kg), Ayacucho (71.63 mg/kg), Cusco (69.37 mg/kg), La Libertad (72.65 mg/kg), and Lima (99.01 mg/kg) exceeded the 50 mg/kg limit established by the Peruvian Environmental Quality Standards. The use of pesticides, including rodenticides, fungicides, insecticides, herbicides, and nematicides, introduces organic and inorganic compounds that may contain metals as part of their formulation. Similarly, both mineral and organic fertilizers can contribute elements such as Cd, Ni, Pb, As, and Cr to the soil, with phosphate-based fertilizers identified as a significant source of Cd contamination [11]. Our results are within the range reported for avocado cultivation soils in Peru, with As concentrations ranging from <LOD to 246 mg/kg (mean: 50.8 ± 58.13 mg/kg) [10]. Similar findings were reported by Guadalupe et al. [11] for coffee soils in the Amazonas region (28.01–98.11 mg/kg), by Bedoya-Perales et al. [68] for potato soils in Moquegua (4.0–182.9 mg/kg), and by Rosales-Huamani et al. [69] for quinoa soils in Huánuco (1.27–3.93 mg/kg), evidencing a heterogeneous yet recurrent distribution of arsenic across different agricultural regions of the country.

Cd remained below the limit of detection (LOD) in most regions, except for Amazonas (0.27 ± 0.21 mg/kg), Ayacucho (0.34 ± 0.05 mg/kg), Ica (0.38 ± 0.86 mg/kg), La Libertad (0.48 ± 0.96 mg/kg), and Lima (0.55 ± 1.04 mg/kg). Cd concentrations in soils from La Libertad (1.58 mg/kg) and Lima (1.86 mg/kg) exceeded the Peruvian Environmental Quality Standards (1.4 mg/kg). In Peru, avocado cultivation soils have been reported to have Cd concentrations ranging from <LOD to 11.98 mg/kg, with an average of 0.54 ± 1.00 mg/kg [10]. These findings indicate a high degree of spatial heterogeneity in Cd accumulation in avocado plantation soils in Peru. The results are consistent with those of Arévalo-Gardini et al. [45], who found lower values in cocoa soils (<LOD–0.21 mg/kg), likely due to the use of phosphate fertilizers, recognized as the main source of Cd in agricultural systems [70,71]. Similar results were reported by Huamaní-Yupanqui et al. [72], who observed Cd accumulation between 0.49 and 0.79 mg/kg, and by Guadalupe et al. [11] in coffee soils (0.07–4.14 mg/kg).

Cr showed one of the greatest regional variabilities, ranging from 0.15 to 66.78 mg/kg. The highest averages were recorded in Ica (38.18 ± 15.56 mg/kg), La Libertad (37.79 ± 18.47 mg/kg), and Lima (35.61 ± 12.96 mg/kg), while the lowest were found in Amazonas (7.46 ± 8.65 mg/kg). The elevated levels in coastal regions may be attributed to industrial discharges, transported sediments, or the use of wastewater for irrigation, whereas the lower levels in high Andean regions reflect less anthropogenic disturbance. However, some samples from Ica (66.78 mg/kg) and Lima (62.89 mg/kg) exceeded the limit, possibly due to residues of herbicides and fungicides containing Cr compounds [15]. Our results fall within the range reported by Solorzano et al. [10] for avocado cultivation soils in Peru, with Cr concentrations ranging from 0.04 to 97.51 mg/kg (mean: 51.52 ± 12.13 mg/kg). Espinoza-Guillén et al. [73] reported comparable concentrations (47.7 mg/kg) in Amazonian soils, while Bedoya-Perales et al. [68] found slightly lower values (1.0–39.8 mg/kg). Guadalupe et al. [11] reported values ranging from 5.24 to 59.33 mg/kg in coffee cultivation soils from northeastern Peru.

Hg concentrations ranged from <LOD to 1.386 mg/kg, showing the greatest regional contrast among the analyzed metals. La Libertad (1.28 ± 0.05 mg/kg) exhibited the highest value, while the other regions showed very low levels (<0.15 mg/kg). These results suggest possible mining influence in La Libertad, as Hg is traditionally used in gold amalgamation. In the remaining regions, Hg levels reflect natural background concentrations. Hg values generally complied with the maximum limit set by the Peruvian Environmental Quality Standards (6.6 mg/kg). Our results are consistent with those reported by Solorzano et al. [10] in Peruvian avocado cultivation soils, which ranged from <LOD to 1.89 mg/kg (mean: 0.17 ± 0.32 mg/kg).

Ni concentrations ranged from 2.48 to 12.78 mg/kg. The highest average was observed in Lima (10.82 ± 4.85 mg/kg), followed by Ayacucho (9.78 ± 2.34 mg/kg). The lowest averages were found in Cusco (4.25 ± 3.25 mg/kg) and Amazonas (4.41 ± 2.48 mg/kg). These results suggest possible anthropogenic inputs in urban and mining-influenced areas. The values were comparable to those reported by Guadalupe et al. [11] (2.37–12.32 mg/kg), Espinoza-Guillén et al. [73] (19.2 mg/kg), and Arévalo-Gardini et al. [45] (1.64–13.69 mg/kg). Although Ni showed regional variability, the observed levels are consistent with its natural occurrence in soils derived from igneous and metamorphic materials. However, the addition of nitrogen fertilizers and organic amendments may also increase its concentrations [19,20,74,75,76].

Pb concentrations ranged from 6.34 to 32.20 mg/kg, with the highest values in Lima (25.35 ± 6.02 mg/kg) and Ayacucho (22.08 ± 7.45 mg/kg). The lowest values were recorded in Huancavelica (8.06 ± 1.97 mg/kg) and Áncash (8.12 ± 1.24 mg/kg). Pb is commonly associated with vehicular emissions, fossil fuel combustion, and industrial waste, which explains its higher presence in Lima and Ayacucho, as these areas are more urbanized or closer to mining zones. Pb levels met the Peruvian Environmental Quality Standards (70 mg/kg); however, their spatial distribution showed significant regional differences. Compared with previous studies, the levels obtained were higher than those reported by Arévalo-Gardini et al. [45] (5.52–15.92 mg/kg) and similar to those observed in Amazonian agricultural soils (16.5 mg/kg) by Espinoza-Guillén et al. [73], as well as those reported by Guadalupe et al. [11] (7.06–34.96 mg/kg).

#### 3.1.2. Concentration of Metals in Avocado

Table 2 summarizes the mean concentrations, standard deviations, and variation ranges of As, Cd, Cr, Hg, Ni, and Pb in avocado fruits from the main producing regions of Peru. Overall, the observed values were low, with most metals below the limit of detection (LOD), indicating a limited translocation from root to the edible portion of the fruit. Plants can absorb metals from soil either passively through water uptake by roots or actively via transport mechanisms across the plasma membrane of epidermal cells [77]. The magnitude of this uptake depends on the physicochemical properties of the soil (pH, organic matter content, texture, and cation exchange capacity) and the physiological characteristics of the plant species [78,79]. Once absorbed, metals can be translocated and accumulated in different tissues [19].

As was detectable only in Ayacucho (0.154 mg/kg), La Libertad (0.125 mg/kg), and Lima (0.152 mg/kg), while concentrations in other regions were below the LOD. In comparison, Castañeda et al. [80] reported mean values of <0.044 mg/kg in Colombia, Mafulul et al. [81] found higher levels (3.53 mg/kg) in Nigeria, and AlJuhaimi et al. [82] detected 3.22 mg/kg in Saudi Arabia.

Cd was detected in trace amounts (<0.03 mg/kg) only in some regions, with average values of 0.011 mg/kg in Ayacucho, 0.016 mg/kg in Ica, 0.028 mg/kg in La Libertad, and 0.033 mg/kg in Lima. The Cd concentrations found in this study fall within the range reported by the National Contaminant Monitoring Program of the Peruvian National Agrarian Health Service (SENASA), whose values between 2022 and 2024 varied from 0.005 to 0.213 mg/kg, with a mean of 0.037 mg/kg. Likewise, the Rapid Alert System for Food and Feed of the European Union (RASFF) reported in 2025 that twelve batches of Peruvian avocados contained Cd levels between 0.05 and 0.10 mg/kg, values that are moderate and consistent with historical patterns documented by SENASA. As illustrated in Supplementary Figure S1, our concentrations align with both datasets, confirming the temporal consistency and stability of Cd levels in Peruvian avocados. These concentrations are similar than those reported by Babuskin et al. [83] (<0.016 mg/kg) in Ethiopia and AlJuhaimi et al. [82] (0.014 mg/kg) in Saudi Arabia, and markedly lower than those found by Mafulul et al. [81] (0.8 mg/kg) in Nigeria.

Cr was below the detection limit (LOD < 0.003 mg/kg) in all fruit samples, confirming that the root acts as an effective physiological barrier for this metal, even in soils with moderate concentrations. Comparable results were reported by Babuskin et al. [83] (0.003 mg/kg), while AlJuhaimi et al. [82] and Mafulul et al. [81] found slightly higher concentrations (0.164 and 0.16 mg/kg, respectively).

Hg concentrations ranged from <0.005 to 0.167 mg/kg, with the highest levels in La Libertad (0.142 mg/kg) and Lima (0.167 mg/kg). The national mean in Peru falls within the range reported in Colombia by Castañeda et al. [80] (0.0386 mg/kg; 0.0045–0.367 mg/kg).

Ni was detected at low levels, ranging from 0.108 to 0.147 mg/kg in La Libertad and Lima, respectively. These values are lower than those reported by Babuskin et al. [83] (3.49 mg/kg) in Ethiopia and slightly higher than those of Mafulul et al. [81] (0.05 mg/kg). The limited accumulation of Ni in the fruit confirms its low translocation capacity in avocado.

Pb was the most frequently detected metal in avocado fruits, with mean concentrations of 0.220 mg/kg in Ayacucho, 0.231 mg/kg in La Libertad, and 0.325 mg/kg in Lima. Castañeda et al. [80] reported 0.149 mg/kg (0.138–0.372 mg/kg) in Colombia, Babuskin et al. [83] found <0.025 mg/kg in Ethiopia, and Mafulul et al. [81] reported 0.02 mg/kg in Nigeria.

### 3.2. Ecological Risk of Metals

#### 3.2.1. Geo-Accumulation Index

**Figure 2:**
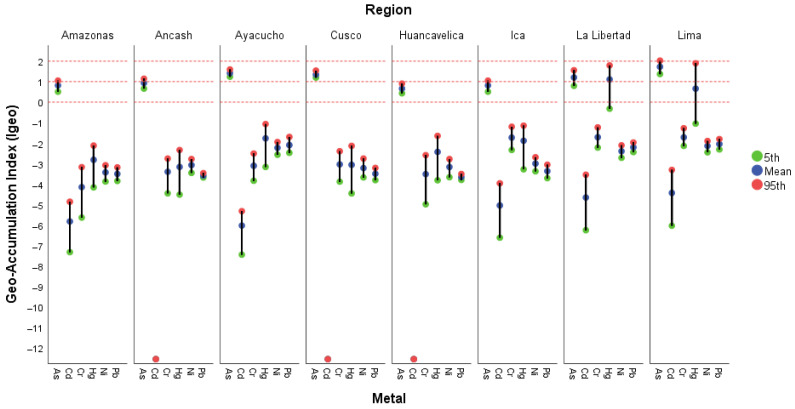
Mean, 5th, and 95th percentile values of the geo-accumulation index (Igeo) per region. Note: Igeo ≤ 0—practically uncontaminated; 0 ≤ Igeo ≤ 1—uncontaminated to moderately contaminated; 1 ≤ Igeo ≤ 2—moderately contaminated; 2 ≤ Igeo ≤ 3—moderately to heavily contaminated; 3 ≤ Igeo ≤ 4—heavily contaminated; 4 ≤ Igeo ≤ 5—heavily to extremely contaminated; and 5 < Igeo—extremely contaminated.

The assessment of soil quality was performed using the geo-accumulation index (Igeo). Figure 2 shows the Igeo values (mean, 5th, and 95th percentiles) for As, Cd, Cr, Hg, Ni, and Pb, categorized by production region (see Table S2). The results indicate that none of the analyzed soils exhibited contamination by Cd, Cr, Ni, or Pb (Igeo < 0), displaying a generally negative trend across all evaluated regions, which suggests non-contaminated conditions. The average Igeo values remained below zero, with slight variations between percentiles. Therefore, it can be inferred that the presence of these metals in soil is of natural origin rather than resulting from anthropogenic activities.

Notable regional differences were observed. In La Libertad and Lima, the mean and 95th percentile values were higher for Hg, and for As across all regions, leading to the classification of soil contamination as ranging from uncontaminated to moderately contaminated (0 ≤ Igeo ≤ 2).

This pattern suggests greater localized metal accumulation and potential anthropogenic contributions associated with urban and industrial activities. In Ayacucho and Cusco, moderate increases in Igeo for As were recorded. In contrast, Amazonas, Áncash, Huancavelica, and Ica exhibited homogeneous values, reflecting natural background conditions and minimal alteration from human activities.

Overall, the results indicate that although most regions exhibit metal accumulation levels within natural ranges, La Libertad and Lima show localized hotspots of Hg enrichment, which could represent a potential site-specific environmental risk.

As shown, the highest Igeo values across all evaluated regions, ranging from uncontaminated to moderately contaminated (0 ≤ Igeo ≤ 2). These values reflect a significant accumulation of As, possibly associated with the use of phosphate fertilizers and arsenical pesticides. Previous studies support this trend: Custodio et al. [13] reported moderate to high contamination levels (2 < Igeo < 4) in the central Andes, and Orellana-Mendoza et al. [84] found values up to 3.15, attributed to anthropogenic influence. Similarly, Santos-Francés et al. [85] reported Igeo values ranging from −3.70 to 3.62 across different regions, with the highest levels in mining areas of Huancavelica and Cajamarca, while Guadalupe et al. [11] found Igeo values <3 in the Amazonas and San Martín regions.

Cd exhibited moderate variability among regions, remaining within the uncontaminated category (Igeo < 0). Higher results were reported by Cáceres-Choque et al. [86], who observed Igeo values ranging from 0 to 3.4 (uncontaminated to heavily contaminated), and by Santos-Francés et al. [85], who found values between −4.57 and 1.63 depending on the study area. In Arequipa, Huerta Alata et al. [87] recorded low average values (0.11 ± 0.59), indicating minimal contamination, while Guadalupe et al. [11] reported values below 1. Overall, the evidence suggests that Cd occurrence is strongly influenced by agricultural management practices and the use of phosphate fertilizers.

For Cr, all evaluated soils exhibited negative Igeo values, indicating no contamination (Igeo < 0). This pattern is consistent with the findings of Espinoza-Guillén et al. [61,73], who obtained values close to zero (0.3 ± 0.1), and with those of Huerta Alata et al. [87], who reported even lower levels (−2.63 ± 0.86). Although some studies, such as Santos-Francés et al. [85], have documented Igeo values up to 3.81 for Cr in mining zones, the agricultural areas assessed in this study show no significant metal accumulation, suggesting a predominantly natural origin.

Ni exhibited negative Igeo values in all regions, indicating that the soils were uncontaminated. The results are consistent with those of Cáceres-Choque et al. [86], who reported values between −1.4 and −0.4, and Espinoza-Guillén et al. [73], who found averages of 0.3 ± 0.1, both indicative of natural levels, as well as with the values below 1 reported by Guadalupe et al. [11]. Santos-Francés et al. [85] reported a wider range (−4.04 to 3.06), with the highest values associated with soils near mining sources. Therefore, Ni does not pose a relevant environmental risk in the evaluated agricultural systems.

Hg exhibited low Igeo values (Igeo < 0) in six of the evaluated regions, indicating no contamination but showing heterogeneous behavior among regions. However, in La Libertad and Lima, the mean and 95th percentile Igeo values ranged from uncontaminated to moderately contaminated (0 ≤ Igeo ≤ 2). Due to Hg’s high toxicity and bioaccumulation potential, even minimal concentrations warrant continuous monitoring, particularly in regions with artisanal mining activities or in soils rich in organic matter, where Hg can be transformed into more bioavailable forms.

Pb displayed negative or near-zero Igeo values across all regions, classifying soils as uncontaminated. These results contrast with those reported by Cáceres-Choque et al. [86], who found values between 0.7 and 3.1 (uncontaminated to highly contaminated), and by Custodio et al. [88], who described slight contamination (0 < Igeo < 1) in the central Andes. Similarly, Santos-Francés et al. [85] reported a wide geographic range (−3.17 to 2.71). In the present study, Pb does not represent a contamination risk, suggesting limited influence from recent anthropogenic sources.

#### 3.2.2. Single Pollution Index

**Figure 3:**
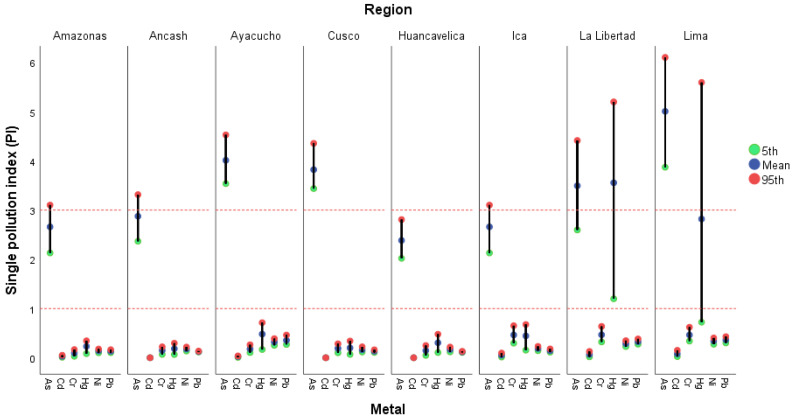
Mean, 5th, and 95th percentile values of the Single pollution index (PI) per region. Note: Absent PI < 1, low level (1 < PI < 2), moderate level (2 < PI < 3), strong level (3 < PI < 5), or high level (PI > 6).

Figure 3 illustrates the behavior of the Individual Contamination Index (ICI) for As, Pb, Cd, Cr, Ni, and Hg. Each point represents the 5th percentile (green), the mean (blue), and the 95th percentile (red), reflecting the variability in metal contamination levels across regions.

In general, most regions exhibit low PI values (below 1), indicating slight or negligible individual contamination by most metals. However, La Libertad and Lima stand out, with mean and 95th percentile PI values considerably higher, approaching or exceeding 5, mainly for As and Hg. This suggests the presence of localized hotspots of more severe contamination in these regions.

Regions such as Ayacucho and Cusco show PI > 3 for As, classifying them as highly contaminated. In contrast, Huancavelica presents the lowest and most homogeneous values, suggesting minimal influence from anthropogenic or industrial sources.

#### 3.2.3. Ecological Risk

**Figure 4:**
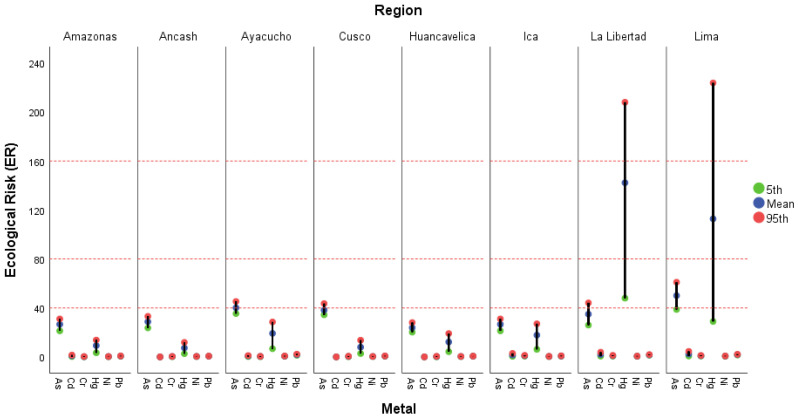
Mean, 5th, and 95th percentile values of the ecological risk (ER) per region. Note: Llow risk (ER ≤ 40), moderate risk (40 < ER ≤ 80), considerable risk (80 < ER ≤ 160), high risk (160 < ER ≤ 320), and very high risk (ER ≥ 320).

Figure 4 shows the variation in ecological risk (ER) associated with As, Cd, Cr, Ni, Hg, and Pb. For each case, the values corresponding to the 5th percentile (green), the mean (blue), and the 95th percentile (red) are presented. Overall, ER values were low across most regions, indicating a low (<40) to moderate (<80) ecological risk. However, for Hg, La Libertad, and Lima exhibited the highest risk values, reaching the high-risk category (160 ≤ ER < 320) at the 95th percentile and the considerable-risk category (80 ≤ ER < 160) at the mean values. This suggests the presence of localized contamination hotspots in these regions with potential for significant ecological impact. In contrast, regions such as Amazonas, Áncash, Huancavelica, and Ica displayed homogeneous and low ER values, with no notable differences between metals or percentiles, suggesting more stable environmental conditions or lower anthropogenic pressure.

The ER values obtained in this study are generally lower than those reported in previous research conducted in Peruvian agricultural soils. For instance, Santos-Francés et al. [85] reported ER values for arsenic (As) ranging from 1.15 to 184.10, encompassing low (<40) to high (>160) risk categories, while cadmium (Cd) showed values between 1.90 and 139.21, corresponding to low to considerable risk levels (80–160). Similarly, Custodio et al. [13] indicated that As exhibited risk levels ranging from moderate to high (70.63–192.19), being one of the elements with the greatest contribution to ecological risk. In the same context, Orellana-Mendoza et al. [43] identified As as the main contributor to total ecological risk (61.5%), followed by Cd and Pb, with an average value of 62.22 ± 32.09. A subsequent study by the same authors in Huancayo reported high to very high risk levels, primarily attributed to As, Cd, and Pb, which also posed threats to human health [75,84].

These findings are consistent with international observations, which consistently identify arsenic and cadmium as the most critical metals in terms of ecological risk and toxicity in agricultural soils [89,90]. Collectively, the results of the present study reflect a relatively more favorable situation compared with previous reports, although continuous monitoring remains necessary—particularly in regions where Hg shows elevated values—to prevent potential long-term cumulative ecological impacts.

### 3.3. Bioaccumulation of Metals

**Figure 5:**
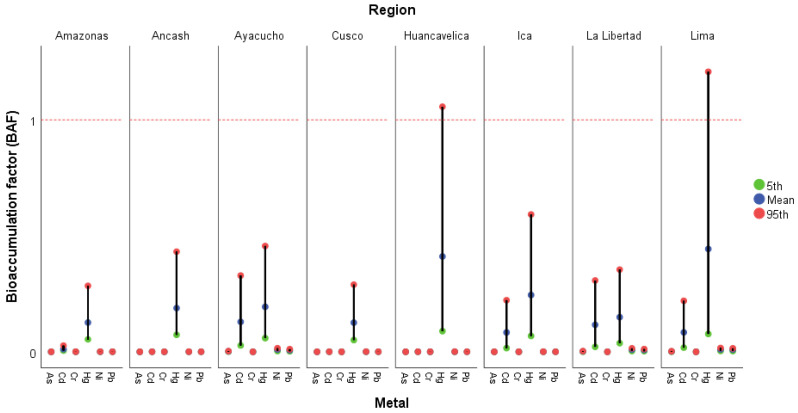
Mean, 5th, and 95th percentile values of the bioaccumulation factor (BAF) per region. Note: BAF < 1 safe, >1 unsafe.

Figure 5 illustrates the behavior of the bioaccumulation factor (BAF) for the evaluated metals (As, Cd, Cr, Hg, Ni, and Pb) in avocados from different producing regions of Peru. In most cases, BAF values are below 1, indicating a low capacity for metal transfer from the soil to the edible fruit. This behavior is consistent with the physiology of the avocado plant, whose root structure and exclusion mechanisms limit the accumulation of potentially toxic elements. However, in the regions of Huancavelica and Lima, greater dispersion and higher values (95th percentile) were observed for Hg, suggesting that under certain environmental or agricultural management conditions, higher bioaccumulation could occur. In contrast, regions such as Amazonas, Cusco, and Ayacucho exhibited the lowest values, reflecting less contaminated soils or lower metal availability for root absorption.

This finding suggests that, in general, avocado has a low capacity to accumulate metals such as As, Cd, Cr, Ni, and Pb, but a potential capacity to accumulate Hg. Metal translocation varies depending on the plant species, plant tissue, total metal content in the soil, and the mobility of each element [91,92]. Overall, the results confirm that metal bioaccumulation in avocado is limited and that the detected levels remain within safe margins. However, the regional variations highlight the need for continued monitoring of agricultural soils and fertilization practices to prevent future accumulations that could compromise fruit safety and export quality.

### 3.4. Health Risk Assessment

#### 3.4.1. Estimated Daily Intake

**Figure 6:**
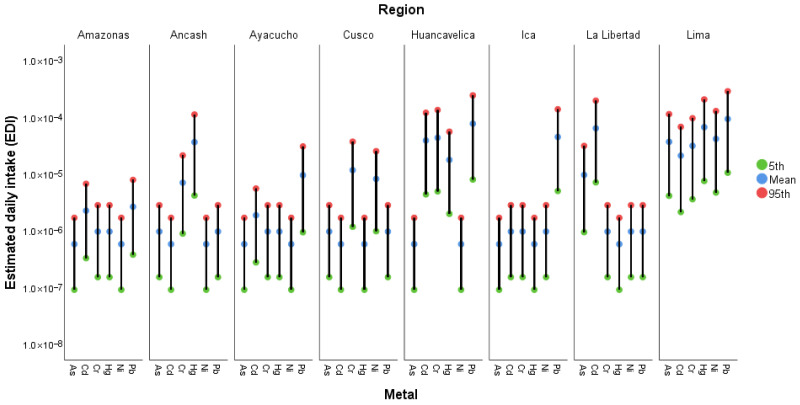
Mean, 5th, and 95th percentile values of the estimated daily intake (EDI) per region.

Figure 6 shows the variation in the estimated daily intake (EDI) of As, Cd, Cr, Hg, Ni, and Pb in avocados from the main producing regions of Peru (see Table S3). The green, blue, and red points represent the 5th percentile, the mean, and the 95th percentile, respectively, obtained through probabilistic simulations. A marked variability among regions is observed, reflecting differences in potential consumer exposure. The regions of Huancavelica, Ica, La Libertad, and Lima exhibit higher mean and 95th percentile values, suggesting greater metal accumulation. In contrast, regions such as Amazonas and Ayacucho show lower exposure levels. Overall, most EDI values fall within the range of 10^−6^ to 10^−5^ mg/kg body weight/day, indicating relatively low exposure.

#### 3.4.2. Hazard Index

**Figure 7:**
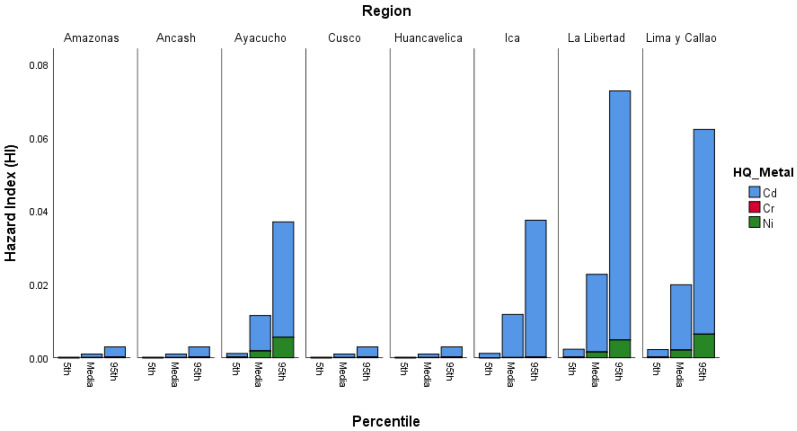
Mean, 5th, and 95th percentile values of the hazard index (HI) per region.

Figure 7 shows the contribution of Cd, Cr, and Ni to the hazard quotient (HQ) and hazard index (HI) across different avocado-producing regions of Peru (see Table S3).

In all cases, HI values remain well below unity (HI < 1), indicating no significant non-carcinogenic risk for consumers under the evaluated conditions. However, a clear regional variability is observed. The regions of Ica, La Libertad, and Lima exhibit the highest HI values, particularly at the 95th percentile, reflecting maximum exposure scenarios or local conditions that promote greater metal accumulation. Cadmium (Cd) is the main contributor to the total HI in all regions, followed by nickel (Ni), while chromium (Cr) shows a minor or negligible influence.

The low HQ values obtained in this study are consistent with those reported in studies conducted on avocado in Ethiopia, where Babuskin et al. [83] reported levels on the order of 10^−5^ for Cr and 10^−4^ for Ni, indicating an insignificant non-carcinogenic risk. However, the values estimated here are considerably lower than those reported in Colombia by Castañeda et al. [80], who documented a non-carcinogenic risk (HQ) of 1.2 for Pb at the 95th percentile, associated with a high ingestion rate (0.200 kg/day, modeled using a lognormal distribution). Similarly, in Nigeria, Mafulul et al. reported elevated HQ values for Cr (16.8) and values close to unity for Cd (0.9) and Ni (0.8), employing an even higher ingestion rate (0.345 kg/day). These differences are mainly explained by the consumption values used in each assessment. In those studies, the estimated intake rates were considerably higher than the average per capita avocado consumption in their respective countries. Since the metal concentrations and body weights applied are comparable to those in the present study, the higher assumed intake is the primary factor driving the observed discrepancies. Consequently, the high HQ values observed may reflect an overestimation of risk derived from dietary intakes that are not representative of the actual population consumption patterns.

#### 3.4.3. Cancer Risk

**Figure 8:**
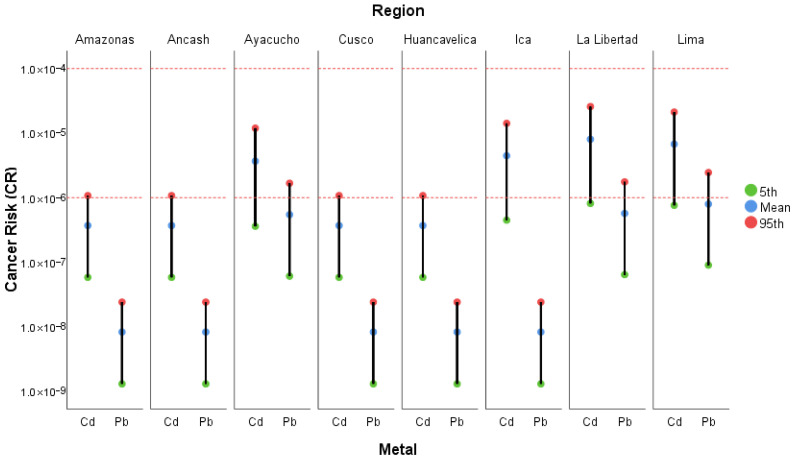
Mean, 5th, and 95th percentile values of the cancer risk (CR) per region. Note: Values < 1 × 10^−6^ indicate no health risk; values between 1 × 10^−6^ and 1 × 10^−4^ represent an acceptable risk; and values > 1 × 10^−4^ reflect a high risk and potential adverse effects.

Figure 8 illustrates the estimated carcinogenic risk (CR) associated with Cd and Pb intake through avocado consumption in the main producing regions of Peru. Overall, CR values for both metals ranged from 10^−8^ to 10^−5^, indicating a low (<10^−6^) to acceptable (10^−4^) carcinogenic risk (see Table S3).

Although the obtained values did not exceed the threshold of concern (1 × 10^−4^), regional variability highlights the need for continuous monitoring of carcinogenic metals, as well as the implementation of sustainable agricultural practices aimed at minimizing the transfer of Cd and Pb from soil to edible fruit. Notably, the regions of Ayacucho, Ica, La Libertad, and Lima exhibited the highest CR values, particularly at the 95th percentile (Figure 8), suggesting that under maximum exposure scenarios, the risk could approach the upper limit of the tolerable range. In contrast, regions such as Amazonas, Áncash, and Huancavelica presented the lowest risk levels. Cd contributed more significantly to CR than Pb, reflecting its higher carcinogenic potential and toxicological relevance in avocado cultivation, underscoring the necessity of its monitoring and control [93,94,95,96,97,98,99].

Similar results have been reported in international studies. For Pb, Mafulul et al. [81] documented mean CR values of 6.63 × 10^−5^, while Castañeda et al. [80] reported a comparable value of 6.43 × 10^−5^, both within the range considered acceptable (10^−6^–10^−4^). In the case of Cd, Mafulul et al. [81] found a mean value of 3.41 × 10^−4^, which approaches the upper limit of the tolerable risk range.

## 4. Conclusions

In this study, the concentration of metals in soils followed the order As > Cr > Pb > Ni > Cd > Hg, whereas in avocado fruits the order was Pb > Hg > As > Ni > Cd, with Cr remaining below detection limits. The differences among regions were statistically significant, with higher values observed in Lima and La Libertad.

The ecological and human health risk assessment under the One Health framework indicated overall low risks. Bioaccumulation factors (BAF < 1) confirmed limited metal translocation. The geo-accumulation index (Igeo) and ecological risk (ER) categorized the soils as uncontaminated to moderately contaminated, with low ecological concern. Regarding human health, hazard quotient (HQ) and hazard index (HI) values < 1 reflected minimal risk for non-genotoxic effects. Cancer risk (CR) values ranged from 10^−8^ to 10^−5^, indicating non-significant carcinogenic risk for Pb (<10^−6^) and an acceptable risk level for Cd (10^−4^).

A key limitation of this study was the inability to analyze the human risks associated with ingestion, inhalation, and dermal exposure to some of the metals studied due to the lack of reference values. Future research should refine these reference bases and expand to other exposure routes.

To mitigate the presence of heavy metals in avocados, farmers are advised to conduct regular soil and water analyses, use certified agrochemicals, and monitor the metal content in products before harvest. Additionally, research can be conducted on the use of avocado varieties that absorb less cadmium, and soil remediation techniques such as phytoremediation can be employed.

## Figures and Tables

**Table 1:** Relevant equations and parameters (Reference: [28,54,55,56,57,58,59,60,61,62]).

Parameter	Abbreviation	Equation or Value	Units
Ecological Risk			
Bioaccumulation factor	BAF	$BAF=C_{avocado\;}/\;C_{soil}$	
Concentration of avocado	$C_{avocado}$	This study	mg/kg
Concentration in soil	$C_{soil}$	This study	mg/kg
Geo-accumulation index	$I_{geo}$	$I_{geo}={log}_{2}\left(\frac{C_{soil}}{1.5B_{n}}\right)$	
Background value	$B_{n}$	As: 15; Cd: 1.0; Cr: 90; Hg: 0.25, Ni: 30; Pb: 70	mg/kg
Ecological risk	*ER*	${ER}_{r}^{i}=T_{r}^{i}\times PI$	
Single pollution index	*PI*	$PI=C_{soil}\;/\;B_{n}$	
Toxicity response coefficient	$T_{r}^{i}$	As: 10; Cd: 30; Cr: 2; Hg: 40, Ni: 2; Pb: 5	
Potential ecological risk index	*RI*	$RI=\sum_{i=1}^{n}ER$	
Health risk			
Estimated daily intake	*EDI*	${(C}_{avocado}\;\times IR)/Bw$)	mg/kgBw/day
Ingestion rate	*IR*	Lognormal (5th: 3.54 × 10^−3^; 50th: 2.38 × 10^−2^; 95%; 7.02 × 10^−2^)	kg/day
Body weight	*Bw*	LogNormal (5th: 45.3; 50th: 62.2; 95th: 85.4)	kgBw
Hazard quotient	*HQ*	EDI / RV	
Reference Value	*RV*	Cd: 0.001; Cr: 1.5; Ni: 0.02	mg/kgBw/day
Hazard index	*HI*	$\sum_{n=1}^{x}{HQ}_{n}$	
Margin of exposure	*MOE*	BMDL_%_/EDI	
Benchmark dose	*BMDL_%_*	BMDL01 (Pb): 1.5 × 10^−3^ (Cardiovascular)	mg/kgBw/day
		BMDL10 (Pb): 6.3 × 10^−4^ (Nephrotoxic)	mg/kgBw/day
Probability of exceedance	*POE*	$Pr\left(EDI>{BMDL}_{x}\right)=\int_{{BMDL}_{\%x}}^{\infty }f(E)\;dE$	
Cancer risk	*CR*	*EDI* × *SF*	
Slope factor	*SF*	Cd: 0.38; Pb: 0.0085	(mg/kgBw/day)^−1^

**Table 2:** Mean, standard deviation (SD), minimum, and maximum concentrations of metals in soil and avocado.

Region	mg/kg	As	Cd	Cr	Hg	Ni	Pb
Soil							
Amazonas	Mean–S.D.	42.414 ± 8.214 ^a^	0.271 ± 0.214 ^a^	7.458 ± 8.657 ^a^	0.075 ± 0.043 ^a^	4.415 ± 2.485 ^a^	9.049 ± 3.007 ^bc^
	Min–Max	29.401–49.611	<LOD–0.618	0.148–18.04	<LOD–0.096	2.487–5.789	6.345–12.578
Ancash	Mean–S.D.	45.114 ± 9.729 ^a^	<LOD	15.304 ± 7.248 ^a^	0.048 ± 0.057 ^a^	5.648 ± 1.514 ^a^	8.124 ± 1.243 ^c^
	Min–Max	31.781–52.471	<LOD	2.568–22.689	<LOD–0.087	3.471–7.048	7.004–9.904
Ayacucho	Mean–S.D.	58.976 ± 7.625 ^a^	0.339 ± 0.056 ^a^	14.963 ± 8.942 ^a^	0.161 ± 0.014 ^a^	9.789 ± 2.348 ^a^	22.081 ± 7.451 ^a^
	Min–Max	49.917–71.631	<LOD–0.414	6.425–27.298	<LOD–0.197	6.589–12.573	16.534–35.978
Cusco	Mean–S.D.	52.674 ± 7.925 ^a^	<LOD	16.428 ± 10.245 ^a^	0.045 ± 0.075 ^a^	4.250 ± 3.247 ^a^	9.245 ± 2.864 ^bc^
	Min–Max	49.911–69.366	<LOD	5.489–29.567	<LOD–0.102	2.973–7.518	6.598–12.240
Huancavelica	Mean–S.D.	34.014 ± 6.504 ^a^	<LOD	12.635 ± 13.425 ^a^	0.088 ± 0.064 ^a^	5.261 ± 2.516 ^a^	8.064 ± 1.974 ^c^
	Min–Max	28.041–45.213	<LOD	0.178–26.751	<LOD–0.138	3.048–7.802	7.189–9.678
Ica	Mean–S.D.	42.414 ± 8.214 ^a^	0.384 ± 0.867 ^a^	38.182 ± 15.564 ^a^	0.140 ± 0.057 ^a^	5.826 ± 2.107 ^a^	10.007 ± 4.614 ^bc^
	Min–Max	29.715–49.216	<LOD–1.178	20.187–66.784	<LOD–0.191	3.671–7.527	6.974–13.746
La Libertad	Mean–S.D.	51.814 ± 18.321 ^a^	0.479 ± 0.964 ^a^	37.789 ± 18.476 ^a^	1.278 ± 0.046 ^a^	8.549 ± 1.932 ^a^	22.217 ± 4.831 ^ab^
	Min–Max	32.784–72.647	<LOD–1.578	23.451–64.789	<LOD–1.386	5.987–11.278	17.726–28.694
Lima	Mean–S.D.	76.174 ± 17.349 ^a^	0.548 ± 1.045 ^a^	35.612 ± 12.960 ^a^	0.367 ± 0.2448 ^a^	10.823 ± 4.850 ^a^	25.348 ± 6.015 ^a^
	Min–Max	50.079–99.012	<LOD–1.864	26.489–62.894	<LOD–1.745	7.215–12.786	19.073–32.203
Avocado							
Amazonas	Mean–S.D.	<LOD	<LOD	<LOD	0.007 ± 0.001 ^a^	<LOD	<LOD
	Min–Max	<LOD	<LOD	<LOD	<LOD–0.008	<LOD	<LOD
Ancash	Mean–S.D.	<LOD	<LOD	<LOD	0.008 ± 0.002 ^a^	<LOD	<LOD
	Min–Max	<LOD	<LOD	<LOD	<LOD–0.010	<LOD	<LOD
Ayacucho	Mean–S.D.	0.154 ± 0.031 ^a^	0.011 ± 0.068 ^a^	<LOD	0.023 ± 0.004 ^a^	0.134 ± 0.018 ^a^	0.220 ± 0.046 ^a^
	Min–Max	<LOD–0.192	<LOD–0.086	<LOD	<LOD–0.029	<LOD–0.146	<LOD–0.272
Cusco	Mean–S.D.	<LOD	<LOD	<LOD	0.005 ± 0.001 ^a^	<LOD	<LOD
	Min–Max	<LOD	<LOD	<LOD	<LOD–0.007	<LOD	<LOD
Huancavelica	Mean–S.D.	<LOD	<LOD	<LOD	0.011 ± 0.003 ^a^	<LOD	<LOD
	Min–Max	<LOD	<LOD	<LOD	<LOD–0.060	<LOD	<LOD
Ica	Mean–S.D.	<LOD	0.016 ± 0.059 ^a^	<LOD	0.020 ± 0.013 ^a^	<LOD	<LOD
	Min–Max	<LOD	<LOD–0.072	<LOD	<LOD–0.041	<LOD	<LOD
La Libertad	Mean–S.D.	0.125 ± 0.021 ^a^	0.028 ± 0.091 ^a^	<LOD	0.142 ± 0.013 ^a^	0.108 ± 0.021 ^a^	0.231 ± 0.046 ^a^
	Min–Max	<LOD–0.159	<LOD–0.130	<LOD	<LOD–0.157	<LOD–0.132	<LOD–0.283
Lima	Mean–S.D.	0.152 ± 0.029 ^a^	0.033 ± 0.098 ^a^	<LOD	0.167 ± 0.0660 ^a^	0.147 ± 0.026 ^a^	0.325 ± 0.073 ^a^
	Min–Max	<LOD–0.184	<LOD–0.120	<LOD	<LOD–0.428	<LOD–0.172	<LOD–0.396

Distinct letters within each column and sample represent statistically significant differences between groups (Tukey test, *p* < 0.05).

## Data Availability

The data and results obtained in this study are included in the article and Supplementary Materials. Any further questions can be addressed to the corresponding authors.

## Supplementary Materials

The following supporting information can be downloaded at: https://www.mdpi.com/article/10.3390/foods15010082/s1, Figure S1: Comparison of Cd concentrations (mg/kg) in Peruvian avocados; Table S1: Validation parameters of the analytical procedure; Table S2: Characterization of bioaccumulation and ecological risk; Table S3: Characterization of health risk.
