# Supplementary material for: Ecological and Human Health Risk Assessment of Metals in Peruvian Avocados Using a Probabilistic Approach
Source: Foods. 2025 Dec 26;15(1):82. doi: 10.3390/foods15010082 (PMC12785639; doi:10.3390/foods15010082)
Supplement: Supplementary file 1 [file foods-15-00082-s001.zip › Table S1.pdf]

Table S1. Validation parameters of the analytical procedure.

Validation Parameters	Heavy metals					
	**Cd**	**Pb**	**Cr**	**Ni**	**As**	**Hg**
LOD* (mg/Kg)	0.005	0.005	0.003	0.005	0.003	0.005
LOQ** (mg/Kg)	0.015	0.015	0.010	0.015	0.010	0.015
Linearity	0.9998	0.9998	0.9994	0.9996	0.9962	0.9835
Working range (mg/Kg)	0-4	0-4	0-4	0-4	0-4	0-5
Recovery (%)	101	99	98	99	99	101

* LOD: Detection limit

** LOQ: Quantification limit
