# Supplementary figures and images for: Ecological and Human Health Risk Assessment of Metals in Peruvian Avocados Using a Probabilistic Approach
Source: Foods. 2025 Dec 26;15(1):82. doi: 10.3390/foods15010082 (PMC12785639; doi:10.3390/foods15010082)

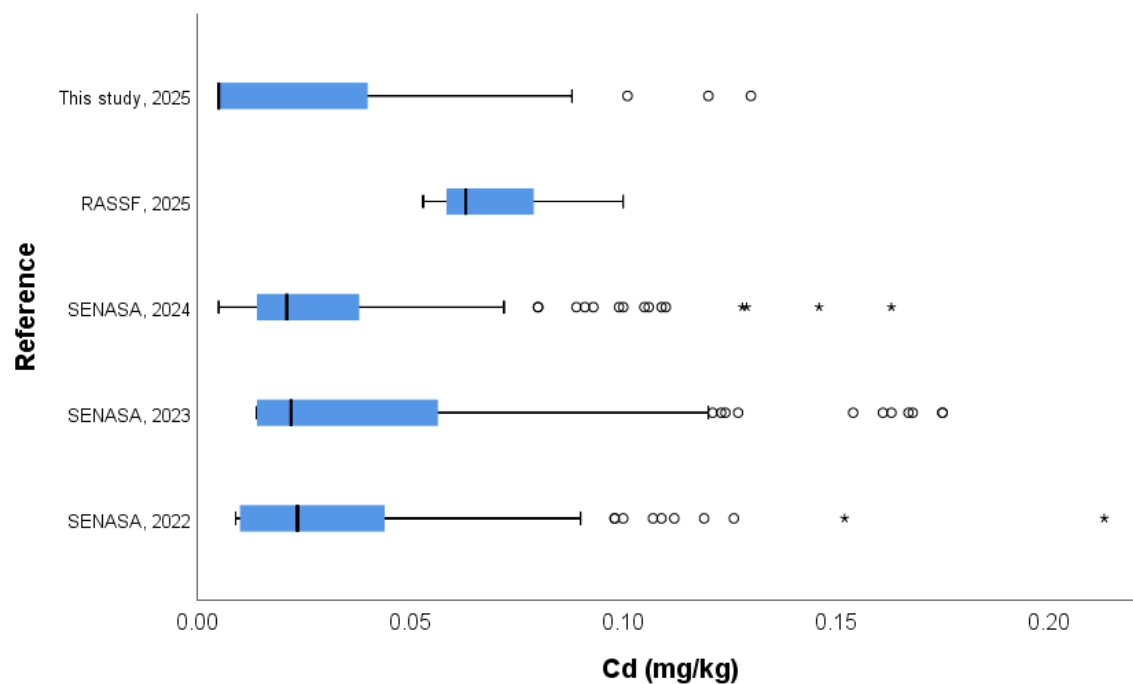

**Figure S1.** Comparison of Cd concentrations (mg/kg) in Peruvian avocados.

Supplement: Supplementary file 1 [file foods-15-00082-s001.zip › Figure S1.pdf]
